# Surveillance perspective on Lyme borreliosis across the European Union and European Economic Area

**DOI:** 10.2807/1560-7917.ES.2017.22.27.30569

**Published:** 2017-07-06

**Authors:** Cees C van den Wijngaard, Agnetha Hofhuis, Mariana Simões, Ente Rood, Wilfrid van Pelt, Herve Zeller, Wim Van Bortel

**Affiliations:** 1 Epidemiology and Surveillance Unit, Centre for Infectious Disease Control, National Institute for Public Health and the Environment (RIVM), Bilthoven, the Netherlands; 2 Epidemiology Unit, KIT (Royal Tropical Institute) Health, Amsterdam, the Netherlands; 3 Office of the Chief Scientist, European Centre for Disease Prevention and Control (ECDC), Stockholm, Sweden; 4 Surveillance and Response Support Unit, European Centre for Disease Prevention and Control (ECDC), Stockholm, Sweden (affiliation when the work was performed); 5 Institute of Tropical Medicine, Antwerp, Belgium (current affiliation)

**Keywords:** surveillance, Europe, Lyme borreliosis

## Abstract

Lyme borreliosis (LB) is the most prevalent tick-borne disease in Europe. Erythema migrans (EM), an early, localised skin rash, is its most common presentation. Dissemination of the bacteria can lead to more severe manifestations including skin, neurological, cardiac, musculoskeletal and ocular manifestations. Comparison of LB incidence rates in the European Union (EU)/European Economic Area (EEA) and Balkan countries are difficult in the absence of standardised surveillance and reporting procedures. We explored six surveillance scenarios for LB surveillance in the EU/EEA, based on the following key indicators: (i) erythema migrans, (ii) neuroborreliosis, (iii) all human LB manifestations, (iv) seroprevalence, (v) tick bites, and (vi) infected ticks and reservoir hosts. In our opinion, neuroborreliosis seems most feasible and useful as the standard key indicator, being one of the most frequent severe LB manifestations, with the possibility of a specific case definition. Additional surveillance with erythema migrans as key indicator would add value to the surveillance of neuroborreliosis and lead to a more complete picture of LB epidemiology in the EU/EEA. The other scenarios have less value as a basis for EU-level surveillance, but can be considered periodically and locally, as they could supply complementary insights.

## Introduction

Lyme borreliosis (LB) is one of the most prevalent vector-borne diseases in Europe. It is caused by *Borrelia burgdorferi* sensu lato bacteria, which in Europe is transmitted by the tick *Ixodes ricinus* [1]. Erythema migrans (EM), an expanding skin rash that occurs around the site of the tick bite, is the most common symptom of early LB [1]. It manifests several days to weeks after the tick bite and can be accompanied by influenza-like symptoms such as fever, headache, mild stiff neck, arthralgia and myalgia. If left untreated, dissemination of the bacteria to other tissues can occur and lead to more severe manifestations that include several skin, neurologic, cardiac, musculoskeletal and ocular manifestations [2]. In Europe neuroborreliosis is the most frequent disseminated manifestation, followed by Lyme arthritis, borrelial lymphocytoma, and, more rarely, acrodermatitis chronica atrophicans and Lyme carditis [2]. Early uncomplicated infection generally responds well to antibiotic treatment, and thus the majority of LB patients have a good prognosis. However, even after repeated antibiotic therapy, depending on the initial clinical presentation, a minority of patients report persisting symptoms such as musculoskeletal pain, neurocognitive symptoms and fatigue [1].

There are differences in LB incidence rates and clinical presentations across European countries, which may partly be due to the heterogeneous distribution of *Borrelia burgdorferi* s.l. genospecies over Europe [3]. Further, the incidence rates of LB across Europe are influenced by geographical, environmental and climatic factors [4-6]. Additionally, human behaviour, including recreational activity, can play a role in LB seasonality [7]. Geographical expansion of the distribution of LB cases has been observed across the European continent [8].

The factors described above are expected to cause true heterogeneity of LB incidence, and thus disease burden, across Europe. However, the heterogeneity found among surveillance systems within Europe complicates the direct comparison of the incidence and trends between countries [9]. LB is a mandatorily notifiable disease in some countries [6]. In countries without mandatory notification, qualified estimates are calculated based on epidemiological studies or incidence estimates from neighbouring comparable countries [2,6]. Under- and over-reporting, as well as differences in case definitions, diagnostic difficulties and different laboratory methods, are recognised issues for LB diagnosis and surveillance [10]. Furthermore, there are differences in data collection (for instance epidemiological surveys vs laboratory based notification systems), and data collection is often not representative of the whole country (e.g. only high-incidence regions are studied). Accordingly, highly divergent incidence rates for LB have been reported between and within some countries.

The objective of the study presented here is to provide a perspective towards more effective and efficient surveillance of LB in the countries in the European Union (EU) and the European Economic Area (EEA). The acquisition of surveillance data in the EU/EEA is needed to assess the importance of LB there, including assessment of disease burden and cost of illness, and will support the prioritisation of public health resources for the prevention and control of LB. We first define relevant characteristics of the LB surveillance system. Secondly, we propose possible surveillance scenarios building on the surveillance characteristics defined and review their advantages, limitations and requirements.

## Surveillance characteristics

Five characteristics of a surveillance system are considered to assess different LB surveillance scenarios in a structured way: (i) the key indicator(s) (e.g. human LB manifestations) being reported, (ii) the reporting entity (e.g. general practitioners (GPs) and/or hospital physicians or laboratories), (iii) coverage of the surveillance used (comprehensive or sentinel), (iv) type of reporting being implemented (mandatory or voluntary notification), and (v) operational level of the surveillance system (national or regional level).

The value and feasibility of different scenarios as indicated by the levels of the surveillance pyramid in the Figure are explored. Within each scenario, the methods of data collection by the five above-mentioned characteristics of surveillance (Table 1) are reviewed, taking into consideration the ease and reliability of the data collection required. These scenarios can be used to make recommendations on the requirements for effective surveillance of LB across Europe. Table 2 reviews the main advantages and limitations of each scenario based upon our assessment.

**Figure:**
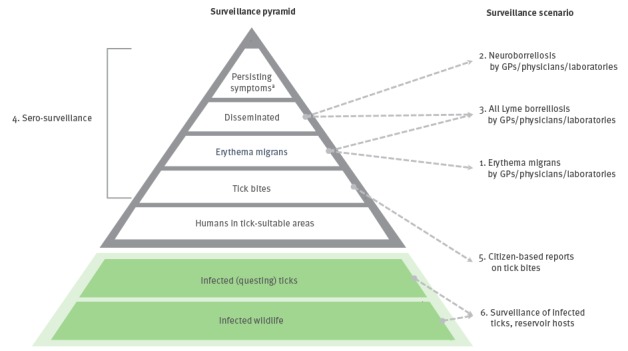
Surveillance pyramid for Lyme borreliosis and six scenarios for surveillance

The different levels of the pyramid embody possible key indicators for Lyme borreliosis surveillance.

GPs: general practitioners.

^a ^Persisting symptoms: Lyme borreliosis (LB)-related persisting symptoms (if developed after a documented episode of LB defined as post-Lyme disease syndrome by the Infectious Diseases Society of America [34]) are long-lasting, often severe and sometimes disabling symptoms that physicians and patients attribute to LB. It remains debated to what extent these symptoms are caused by a current or preceding *Borrelia* infection. Surveillance pyramid adapted from [43].

**Table 1:** The five surveillance characteristics considered for the European Union/European Economic Area perspective on Lyme borreliosis surveillance

Characteristic	Category	Features
**1. Key indicators according to the ** **Figure**	Erythema migrans and disseminated infections	- Surveillance of the different stages of LB manifestations enables assessment of LB incidence in humans. - Surveillance of more than one LB manifestation allows for the estimation of the relative proportions at which the different LB manifestations occur.
	Humans in tick-suitable areas and tick bites	- Determination of exposed groups i.e. humans in tick-suitable areas, from which a subgroup will acquire tick bites.
	Infected wildlife and ticks	- Determination of the dynamics of pathogen species in reservoir and tick populations.
**2. Reporting entity**	GP, other physicians and laboratories	- Reporting of human cases. - Laboratories can provide data on *Borrelia*-specific antibodies for seroprevalence studies. Since patients with diagnostic requests for LB will not necessarily be representative of the general population, clinical data should also be included; diagnostic tests are often requested for patients without specific symptoms for LB, and the seroprevalence in this group of patients can give a preliminary indication of the level of seroprevalence in the general population.
	Research groups	- Ecological studies can provide data on tick and reservoir abundance as well as infection rates with *Borrelia* spp.
	General public	- Direct reports on human exposure to tick bites can be collected through citizen science (i.e. the public self-reporting tick bites).
**3. Comprehensive or sentinel surveillance**	Comprehensive surveillance	- Allows for the estimation of incidence rates and identification of patterns and trends at national and regional level. - Not only structural reporting by hospitals, GPs/other physicians and other health workers, but also periodic surveys can be considered, such as the Dutch GP surveys [12-15]. - To limit under-reporting, often only a limited number of parameters or indicators are collected. - Under-reporting can occur due to complicated reporting systems, lack of perceived benefit, or high workload and/or competing tasks for healthcare workers.
	Sentinel surveillance	- Definition of the catchment populations is crucial. It can differ substantially (in size and accuracy) between countries due to the healthcare system in place. - Reporting entities of sentinel surveillance often agree to participate and are specifically trained, therefore data produced may be expected to be more timely and detailed. - A certain proportion of the cases are studied, allowing for a more intensive investigation of each case. - Data collected may not be representative of the whole country. - Well-designed sentinel systems can efficiently detect national or European trends and monitor the burden of disease using limited resources but may be insensitive to rare events, which can occur outside the catchment population and/or geographical areas included in the sentinel system [38]. - When comparing surveillance data collected through comprehensive or sentinel surveillance, differences in under-reporting should be taken into account.
**4. Mandatory or voluntary notification**	Mandatory reporting	- Direct estimation of incidences by relating the reported counts to the catchment population sizes, which are known and available per country or region. - Under-reporting is observed for many mandatorily notifiable diseases in Europe [39,40]. - Mandatory reporting demands a high level commitment of healthcare professionals. - It may result in lower quality collected data than expected due to either lack of motivation or the cumbersomeness of the reporting procedure [39]. - Motivation to report may be different between countries and depends largely on the awareness of physicians for LB. Other factors such as workload, perceived severity of the disease and knowledge of the obligation to notify might also influence the reporting.
	Voluntary reporting	- Compliance possibly higher due to higher motivation and involvement of reporting entities. - The information collected is expected to have higher quality but it would be difficult to obtain data from the whole country [41]. - The catchment population of the reporting entities should be known to accurately estimate the incidence of LB. - When comparing surveillance data collected through voluntary and/or mandatory reporting, differences in under-reporting between systems should be taken into account, i.e. under-reporting should be measured and corrected for [42].
**5. Surveillance administrative level**	National level	- Allows for a comparison between countries. - Reflects the burden of disease within a country but does not account for regional differences in the incidence of disease.
	Regional level	- Provides information at low administrative level allowing for comparisons between regions or counties. - May not be representative of the national picture if only hotspots are monitored [4,6,9].

GP: general practitioner; LB: Lyme borreliosis.

**Table 2:** Summary of the main characteristics, advantages, limitations and requirements of the six proposed scenarios for surveillance of Lyme borreliosis across the European Union/European Economic Area

Surveillance Scenario	Key indicator for surveillance	Who is reporting	Advantages	Limitations	Requirements
1	Erythema migrans	Hospital physicians/GPs	- Relatively easy recognised and diagnosed, without the need for laboratory confirmation. - Most common manifestation of early LB. - More information on neuroborreliosis multiplication factor^a^.	- Motivation to report may be low for this mild condition. - Probability of clinically diagnosing EM may differ between low- and high-incidence countries or regions. - In some countries GP/other physicians’ catchment populations may be difficult to achieve or estimate.	- In each country GPs/other physicians should be reached and motivated to report EM cases. - Accurate administrations of the names and addresses of GPs/other physicians would facilitate this process. - Communication campaigns can be launched by national or regional public health officials. - In case of not comprehensive surveillance, for each reporting GP/physician their catchment population needs to be known (or estimated) to be able to calculate accurate incidence rates.
2	Neuroborreliosis	Laboratories Hospital physicians/GPs	- Precise and standardised case definition possible, building upon the EFNS guidelines [22]. - One of the most frequent manifestations of disseminated LB, and because of its severity possibly less susceptible to under-reporting. - In some countries reporting based on laboratory information systems may even further reduce under-reporting.	- Laboratory diagnostics on CSF not standard in all countries. - Little data on multiplication factor to other manifestations, which may differ between countries because of the heterogeneous distribution of *Borrelia* genospecies [2-4,22]. - Less sensitive to trends in time than EM because of its lower occurrence rate.	- National and regional laboratories and/or GPs/other physicians should be able to report cases based upon standardised case definitions. - In case of not comprehensive surveillance, the catchment population of the reporting entities must be known and representative of the total population. - Starting by one or a few central laboratories per country may be sufficient to have standardised and comparable data between countries.
3	All LB manifestations	Laboratories, hospital physicians/GPs	- Incidence estimated for the complete spectrum of LB. - Complete information on neuroborreliosis multiplication factor. - Will facilitate assessment of the disease burden of LB in DALYs (e.g. healthy life-years lost), to allow policymakers to compare the impact of LB with other (infectious) diseases [11,31].	- Surveillance of all LB manifestations will have a huge reporting burden. For countries with a high incidence of LB, notification of all cases will not be feasible because the workload would be too high for physicians - Diagnosing the disseminated manifestations of LB (other than neuroborreliosis) can be complicated, resulting in a high risk of inconsistencies (and a risk of lack of specificity) in surveillance data. - Standardisation of all LB manifestations might be difficult. - Cumbersomeness of assessing all laboratory and clinical criteria per patient may result in under-reporting. - High costs will be involved in training of personnel, and extensive quality control would be needed to guarantee representativeness and compatibility between countries.	- In each country GPs/other physicians, complemented by national and regional laboratories, should be reached and motivated to report all LB cases. - Accurate administrations of the names and addresses of GPs/other physicians would facilitate this process. - Communication campaigns can be launched by national or regional public-health officials. - In case of not comprehensive surveillance, for each reporting GP/other physician their catchment population needs to be known (or estimated) to be able to calculate accurate incidence rates.
4	Seroprevalence	Population-based studies, laboratories, GPs	- No under-reporting because seroprevalence studies are not dependent on reporting by other entities than laboratories. - Standardisation of laboratory criteria possible in a prospective setting. - Seroprevalence studies provide additional epidemiological data, such as information on risk factors and spatial patterns that can be used to complement data from other notification systems [17,28].	- Only seroprevalence (historical exposure) can be measured, and no data on the incidence of LB (active infection) can be derived. - Neither new cases that have emerged recently nor the real disease burden can be assessed through such studies [27]. - In a prospective setting, a complete and representative sampling for this purpose can be expensive. - Different serological tests are available targeting different antibodies and having different sensitivities and specificities. Standardisation among countries is needed if inter-country comparisons is aimed for.	- Careful design of a seroprevalence study is required to obtain a representative sample of all regions of the country. - There is the need to clearly define and standardise the laboratory methodology and criteria across countries for comparability.
5	Tick bites	General public	- Hotspots of human exposure to tick bites can be detected with relatively high sensitivity, which can be used to steer regional intervention strategies. - National and regional communication campaigns will improve awareness of the public and physicians of LB. - Characteristics of hotspots can also be compared between countries, and be used as input in a knowledge-based European risk map.	- Awareness and education of the public is needed to generate a sufficient report rate of tick bites. - The number of tick bite reports over time will be influenced by media attention and thus not always accurately reflect temporal trends of tick bites or LB. - Media attention for online reporting of tick bites will differ between countries, which will complicate quantitative comparison between countries.	- Public awareness of the risk for LB is needed as a motivation to report tick bites, which requires national and regional media campaigns to inform the public about online reporting. - Communication of the results is needed to inform and motivate the participants.
6	Tick or reservoir hosts *Borrelia* prevalence	Research groups	- Complementary to human LB surveillance. - Results would improve the prospective surveillance of LB by providing more insight on the ecological and epidemiological features of LB. - Periodic research studies are already standard in many countries.	- It is a complicated process to timely collect catchment data with substantial coverage in a relatively standardised manner. - Data on the tick/reservoir *Borrelia* prevalence is not necessarily associated with the number of LB cases in humans. - Newly invaded tick areas may be missed. These possibly contribute more to increased tick bite risk than established catchment areas and will have a different temporal trend.	- A European network is needed to standardise sampling and collection protocols and to gather national catchment data on ticks and reservoirs from the national and regional networks that already perform such surveillance. - VectorNet^b^ could possibly facilitate future integration and comparison of these data [33]. - It has already been shown that based on the currently available data in published literature space and time trends of infected tick can be assessed.

DALY: disability-adjusted life year; EFNS: European Federation of Neurological Societies; EM: erythema migrans; GP: general practitioner; LB: Lyme borreliosis.

^a^ The factor (or factors) permitting estimation of the incidence of all LB manifestations based on surveillance data from another LB manifestation, e.g. estimate the incidence of neuroborreliosis based upon the incidence of EM.

^b^ VectorNet is a joint initiative of the European Food Safety Authority (EFSA) and the European Centre for Disease Prevention and Control (ECDC). The project supports the collection of data on vectors related to both animal and human health in Europe and the Mediterranean basin: http://ecdc.europa.eu/en/healthtopics/vectors/VectorNet/Pages/VectorNet.aspx

## Surveillance scenarios

### Scenario 1: Erythema migrans as key indicator

EM is the most common manifestation of LB, and the only manifestation in which the clinical symptoms are characteristic of the disease, although not all cases of EM will be diagnosed, especially not if cases present with an atypical skin lesion [2]. It can be monitored through GPs and/or other physicians reporting clinical EM diagnoses, without the need for laboratory confirmation or interpretation (Table 2). Data can be collected through repeated cross-sectional retrospective surveys, as has been implemented in the Netherlands and some other countries by means of GP surveys [5,11-15]. Other options would be implementation through prospective or retrospective sentinels among hospitals and GP practices [16-18]. These options work mainly on a voluntary basis and are shown to provide high response rates and good regional and national estimates to identify temporal and spatial trends [12]. A nationwide approach would be preferable, including, if possible, regional data collection to identify regional LB transmission hotspots.

### Scenario 2: Neuroborreliosis as key indicator

Neuroborreliosis is one of the most frequent manifestations of disseminated LB [2,19]. Mandatory reporting of neuroborreliosis is already common practice in some countries [6,9,17,19-21].

According to the European Federation of the Neurological Societies (EFNS) guidelines for confirmed cases, neurological symptoms have to be laboratory-confirmed by cerebrospinal fluid (CSF) pleocytosis and intrathecal specific antibody production [22]. Compliance with this set of laboratory criteria can, however, be cumbersome. For instance, the Danish microbiology database (MiBa) for neuroborreliosis does not include information on pleocytosis, and, with cases defined as individuals with intrathecal specific antibody production, had a higher capture rate of cases and a more timely data collection than the mandatory notification system in Denmark [20]. Therefore, it may be advisable to allow some levels of confidence for case classification: e.g. possible/probable/confirmed cases. Some countries already use such case classification [23]. In countries where electronic laboratory reporting systems are available, implementation of nationwide laboratory surveillance for neuroborreliosis could be feasible.

Surveillance can also be based on reporting by physicians, especially in countries where testing of CSF is not standard and/or laboratory information systems do not facilitate reporting. However, the information collected in this way will need to be interpreted with caution due to uncertainties in the diagnosis and comparability between countries.

At lower administrative levels or in a sentinel setting, surveillance of neuroborreliosis may be relatively insensitive, since neuroborreliosis is a relatively rare outcome compared with EM, occurring in 3–38% of LB cases, whereas EM occurs in 60–95% of all diagnosed LB manifestations [11,16,17,24] (Table 2).

### Scenario 3: All manifestations of Lyme borreliosis as key indicator

Manifestations other than EM and neuroborreliosis are often more difficult to diagnose. If implemented in a surveillance system, case definitions should be based both on clinical and laboratory diagnostic criteria [2,25]. Data collection can be performed through reporting GPs or other physicians and complemented by laboratory reporting e.g [5,11,16]. Nationwide and comprehensive surveillance systems, when compared with regional and sentinel approaches, will provide better information on the proportion of the occurrence of each LB manifestation (Table 2). The reporting entities can be invited to participate voluntarily in such surveillance; mandatory reporting of all LB cases may result in under-reporting, especially if GPs and other physicians are requested to include specific laboratory criteria in their assessment of possible cases [10,26,27]. For countries with a high incidence of LB, notification of all cases is not feasible because the workload may be too high for physicians and public health authorities.

### Scenario 4: Seroprevalence as key indicator

Seroprevalence studies for *Borrelia*-specific antibodies can be conducted at population level, either by using biobanks or blood donors’ samples or by collecting samples prospectively. These studies can be performed nationally, to give an overall picture of the exposure to *Borrelia* within the country and allow for comparison with other EU countries if standard laboratory methods are used (Table 2). Laboratories can also be motivated to supply available laboratory datasets on tests carried out for diagnostic purposes, to give a first indication about the possible seroprevalence in a country, especially if clinical data is available as well. Nevertheless, for a valid estimate of the seroprevalence in a country or region specifically designed seroprevalence studies are needed on a representative sample of the general population. During such studies additional epidemiological data can also be collected, such as information on risk factors and spatial patterns, which complement the data from other notification systems [17,28]. However, the data reveal a cumulative incidence proportion of exposure to *B. burgdorferi* s.l. and not the incidence of new disease [28].

### Scenario 5: Reported tick bites as key indicator

The spatial distribution of tick bites might reflect human exposure to the risk of LB. A relatively new approach for such spatial surveillance is online citizen-based reporting of tick bites, as has been implemented e.g. in the Netherlands from 2006 to 2011 through www.natuurkalender.nl, and since 2012 through www.tekenradar.nl, in Belgium through TekenNet https://tekennet.wiv-isp.be/ or in Switzerland through https://zecke-tique-tick.ch/en/tickbite-map-switzerland/. A similar follow-up of hotspots where people have greatest exposure to infected ticks can be implemented in other European countries (Table 2). In addition, regional infection rates of ticks removed from humans can be obtained by having the ticks sent to national laboratories. Furthermore, the risk of and risk factors for LB after a tick bite can be assessed by online follow-up of participants to report subsequent development of LB. This is preferably followed by confirmation of the LB diagnosis through the diagnosing physician. Such prospective studies can be temporarily added to complement routine surveillance.

### Scenario 6: Infected tick/reservoir hosts as key indicator

Europe-wide surveillance and/or studies of tick or reservoir could be complementary to human LB surveillance. Periodic (e.g. monthly) catchment data on ticks and reservoirs can be collected as standardised as possible to derive their prevalence of occurrence and *Borrelia* spp. infection rates (Table 2). Results would improve prospective surveillance of LB in the focus area by providing more insight into the ecological and epidemiological features of LB and allows identification of the different genospecies circulating in an area [17,29,30].

## Conclusions

To facilitate better assessment of temporal and spatial trends of LB incidence in Europe, a more standardised surveillance and reporting procedures of LB is required. Priority should be given to standardisation of the key indicators that are put under surveillance between EU countries.

Neuroborreliosis surveillance (scenario 2) across the EU/EEA seems the most feasible and useful scenario, as neuroboreliosis is one of the most frequent severe manifestations. The implementation of a standardised EU case definition is feasible from an operational perspective. Nevertheless, consensus on a standardised case definition is needed between the EU/EEA countries, possibly with some levels of confidence for case classifications e.g. probable/confirmed cases because of difficulties in complying with the laboratory criteria. Surveillance of neuroborreliosis across the EU/EEA will allow for a more accurate assessment of temporal and spatial trends, and the epidemiological features and disease burden of neuroborreliosis. Although complete standardisation would, in theory, lead to the highest comparability of surveillance data, it is probably not feasible to standardise all characteristics of surveillance over all EU/EEA countries. Depending on the country, it will be feasible and cost-efficient to implement surveillance through: (i) laboratory information systems or GPs/other physicians; (ii) comprehensive or sentinel surveillance, although the latter might not be sensitive enough; (iii) mandatory or voluntary reporting, and/or (iv) regional or national reporting. This heterogeneity of feasible surveillance characteristics is caused by dissimilarities in healthcare data logistics (e.g. presence or absence of a national laboratory information system that can be used to detect cases; presence or absence of representative GP sentinels) and the different structures of countries’ healthcare systems (e.g. presence or absence of an exclusive role for GPs for all primary care). Based on the above-mentioned surveillance characteristics, each country should consider how best to achieve valid neuroborreliosis incidence reports that meet EU-wide surveillance criteria. Internal validation studies should be periodically conducted to assess and correct for under- and over-reporting, to allow comparison between countries.

In addition to surveillance of neuroborreliosis, countries can consider surveillance of EM (scenario 1), depending on the local feasibility of implementation. EM comprises a relatively simple diagnosis with no laboratory confirmation needed and can be easily reported by physicians. Nevertheless, also for EM surveillance, the above-mentioned differences in healthcare system and data logistics should be taken into account; e.g. in countries where GPs have an exclusive role in primary care, surveillance of EM can be based on GP sentinels or surveys, whereas in other countries other physicians should also be included in surveillance. The key advantage of surveillance of both EM and neuroborreliosis is that it allows estimation of country-specific multiplication factors between these manifestations, which may discern effects of the *Borrelia* genospecies prevalence on the relative proportions of neuroborreliosis and EM. This may lead to a much closer and more complete picture of the epidemiology and disease burden of LB, and a higher sensitivity for temporal and spatial trends.

Reporting of all LB manifestations and seroprevalence studies (scenarios 3 and 4, respectively) does not seem feasible or effective as a form of permanent surveillance. Nevertheless, periodic seroprevalence studies, despite the limitations observed, can be used to relate human exposure to the incidence of LB [28]. To limit the costs, these should be incorporated into (international) seroprevalence studies of other infectious diseases. Furthermore, there would be additional value in assessing the relative proportions of all LB manifestations with a periodicity of e.g. 5–10 years within each country or region [11]. These relative proportions can be used to derive multiplication factors to estimate the incidence of all LB manifestations based upon the permanent neuroborreliosis and/or EM surveillance data. Such incidence estimates of the complete range of LB manifestations would also facilitate expressing the disease burden of LB in disability-adjusted life years (DALYs) (e.g. healthy life-years lost), and cost-of-illness, to allow policymakers to compare with other infectious and non-infectious diseases [11,31,32].

Additional sources of information can be citizen-based reporting of tick bites and surveillance and/or studies of tick abundance and infection rates. Tick data can be periodically analysed to derive temporal and spatial trends of tick abundance and infection rates (scenario 6). The online reporting of tick bites in the Netherlands has revealed spatial and seasonal patterns of human exposure to tick bites (scenario 5). VectorNet, the joint initiative of the European Food Safety Authority (EFSA) and the European Centre for Disease Prevention and Control (ECDC) that supports the collection of data on vectors related to both animal and human health in Europe and the Mediterranean basin, could possibly facilitate future integration and comparison of data on tick abundance and seasonality [33].

LB-related persisting symptoms (if developed after a documented episode of LB defined as post-Lyme disease syndrome by the Infectious Diseases Society of America [34]) is a growing concern, underlined by a survey in the Netherlands where annual incidence was estimated at 5.5 new cases per 100,000 population in 2010, and disease burden at 86% of the total burden due to LB [11,31]. It concerns long-lasting, often severe and sometimes disabling symptoms that physicians and patients attribute to LB [34]. However, it remains debated to what extent these symptoms are caused by a current or preceding *Borrelia* infection [2,35,36]. As a result, routine surveillance of the syndrome would not provide accurate and reliable data on which response actions can be designed, which is the reason why this is not included as a scenario. Specific studies should first provide more insight into the mechanisms that cause these persisting symptoms [36,37].

In conclusion, surveillance of Lyme neuroborreliosis seems to be the most appropriate way to initiate LB surveillance in the EU despite difficulties to comply with the laboratory criteria. Additional surveillance or specific surveys of the incidence of EM would be of great added value and could possibly be undertaken in sentinel sites. Surveillance and monitoring of the other key indicators would lead to an even more complete picture of temporal and spatial trends, epidemiological features and disease burden of LB, but are more appropriate for specific surveys than for routine surveillance.

## References

[1] Stanek G Wormser GP Gray J Strle F Lyme borreliosis. Lancet. 2012;379(9814):461-73. 10.1016/S0140-6736(11)60103-7 21903253

[2] Stanek G Fingerle V Hunfeld KP Jaulhac B Kaiser R Krause A Lyme borreliosis: clinical case definitions for diagnosis and management in Europe. Clin Microbiol Infect. 2011;17(1):69-79. 10.1111/j.1469-0691.2010.03175.x 20132258

[3] van Dam AP Kuiper H Vos K Widjojokusumo A de Jongh BM Spanjaard L Different genospecies of Borrelia burgdorferi are associated with distinct clinical manifestations of Lyme borreliosis. Clin Infect Dis. 1993;17(4):708-17. 10.1093/clinids/17.4.708 7903558

[4] Lindgren E, Jaenson TGT. Lyme borreliosis in Europe: influences of climate and climate change, epidemiology, ecology and adaptation measures. Copenhagen: World Health Organization Europe; 2006. Available from: http://www.euro.who.int/__data/assets/pdf_file/0006/96819/E89522.pdf

[5] Cimmino MA Relative frequency of Lyme borreliosis and of its clinical manifestations in Europe. European Community Concerted Action on Risk Assessment in Lyme Borreliosis. Infection. 1998;26(5):298-300. 10.1007/BF02962251 9795788

[6] Hubálek Z Epidemiology of lyme borreliosis. Curr Probl Dermatol. 2009;37:31-50. 10.1159/000213069 19367096

[7] Dobson AD Taylor JL Randolph SE Tick (Ixodes ricinus) abundance and seasonality at recreational sites in the UK: hazards in relation to fine-scale habitat types revealed by complementary sampling methods. Ticks Tick Borne Dis. 2011;2(2):67-74. 10.1016/j.ttbdis.2011.03.002 21771540

[8] Medlock JM Hansford KM Bormane A Derdakova M Estrada-Peña A George JC Driving forces for changes in geographical distribution of Ixodes ricinus ticks in Europe. Parasit Vectors. 2013;6(1):1. 10.1186/1756-3305-6-1 23281838 PMC3549795

[9] Smith R Takkinen J Lyme borreliosis: Europe-wide coordinated surveillance and action needed? Euro Surveill. 2006;11(6):E060622.1. 16819127 10.2807/esw.11.25.02977-en

[10] European Centre for Disease Prevention and Control (ECDC). Meeting report. Second expert consultation on tick-borne diseases with emphasis on Lyme borreliosis and tick-borne encephalitis: Stockholm, Sweden, 22–23 November 2011. Stockholm: ECDC; 2012. Available from: http://www.ecdc.europa.eu/en/publications/Publications/Tick-borne-diseases-meeting-report.pdf

[11] Hofhuis A Harms M Bennema S van den Wijngaard CC van Pelt W Physician reported incidence of early and late Lyme borreliosis. Parasit Vectors. 2015;8(1):161. 10.1186/s13071-015-0777-6 25889086 PMC4363353

[12] Hofhuis A Harms M van den Wijngaard C Sprong H van Pelt W Continuing increase of tick bites and Lyme disease between 1994 and 2009. Ticks Tick Borne Dis. 2015;6(1):69-74. 10.1016/j.ttbdis.2014.09.006 25448421

[13] Hofhuis A van der Giessen JW Borgsteede FH Wielinga PR Notermans DW van Pelt W Lyme borreliosis in the Netherlands: strong increase in GP consultations and hospital admissions in past 10 years. Euro Surveill. 2006;11(6):E060622.2. 16819128 10.2807/esw.11.25.02978-en

[14] den Boon S Schellekens JF Schouls LM Suijkerbuijk AW Docters van Leeuwen B van Pelt W Verdubbeling van het aantal consulten voor tekenbeten en Lyme-borreliose in de huisartsenpraktijk in Nederland. [Doubling of the number of cases of tick bites and lyme borreliosis seen by general practitioners in the Netherlands]. Ned Tijdschr Geneeskd. 2004;148(14):665-70. Dutch. 15106318

[15] de Mik EL van Pelt W Docters-van Leeuwen BD van der Veen A Schellekens JF Borgdorff MW The geographical distribution of tick bites and erythema migrans in general practice in The Netherlands. Int J Epidemiol. 1997;26(2):451-7. 10.1093/ije/26.2.451 9169184

[16] Vandenesch A Turbelin C Couturier E Arena C Jaulhac B Ferquel E Incidence and hospitalisation rates of Lyme borreliosis, France, 2004 to 2012. Euro Surveill. 2014;19(34):20883. 10.2807/1560-7917.ES2014.19.34.20883 25188613

[17] Wilking H Stark K Trends in surveillance data of human Lyme borreliosis from six federal states in eastern Germany, 2009-2012. Ticks Tick Borne Dis. 2014;5(3):219-24. 10.1016/j.ttbdis.2013.10.010 24656810

[18] Vanthomme K Bossuyt N Boffin N Van Casteren V Incidence and management of presumption of Lyme borreliosis in Belgium: recent data from the sentinel network of general practitioners. Eur J Clin Microbiol Infect Dis. 2012;31(9):2385-90. 10.1007/s10096-012-1580-3 22391757

[19] Christova I Komitova R Clinical and epidemiological features of Lyme borreliosis in Bulgaria. Wien Klin Wochenschr. 2004;116(1-2):42-6. 10.1007/BF03040423 15030123

[20] Dessau RB Espenhain L Mølbak K Krause TG Voldstedlund M Improving national surveillance of Lyme neuroborreliosis in Denmark through electronic reporting of specific antibody index testing from 2010 to 2012. Euro Surveill. 2015;20(28):21184. 10.2807/1560-7917.ES2015.20.28.21184 26212143

[21] Lopes de Carvalho I Núncio MS Laboratory diagnosis of Lyme borreliosis at the Portuguese National Institute of Health (1990-2004). Euro Surveill. 2006;11(10):257-60. 17130658

[22] Mygland A, Ljøstad U, Fingerle V, Rupprecht T, Schmutzhard E, Steiner I. EFNS guidelines on the diagnosis and management of European Lyme neuroborreliosis. Eur J Neurol. 2010;17(1):8-16, e1-4. 10.1111/j.1468-1331.2009.02862.x 19930447

[23] Paradowska-Stankiewicz I Chrześcijańska I Lyme disease in Poland in 2012. Przegl Epidemiol. 2014;68(2):275-7, 375-7. 25135514

[24] Dryden MS Saeed K Ogborn S Swales P Lyme borreliosis in southern United Kingdom and a case for a new syndrome, chronic arthropod-borne neuropathy. Epidemiol Infect. 2015;143(3):561-72. 10.1017/S0950268814001071 24814098 PMC9507055

[25] Leeflang MM Ang CW Berkhout J Bijlmer HA Van Bortel W Brandenburg AH The diagnostic accuracy of serological tests for Lyme borreliosis in Europe: a systematic review and meta-analysis. BMC Infect Dis. 2016;16(1):140. 10.1186/s12879-016-1468-4 27013465 PMC4807538

[26] Rizzoli A Hauffe H Carpi G Vourc HG Neteler M Rosa R Lyme borreliosis in Europe. Euro Surveill. 2011;16(27):19906. 21794218

[27] Muller I, Freitag MH, Poggensee G, Scharnetzky E, Straube E, Schoerner C, et al. Evaluating frequency, diagnostic quality, and cost of Lyme borreliosis testing in Germany: a retrospective model analysis. Clin Dev Immunol. 2012;2012:595427. 10.1155/2012/595427 PMC3254124 22242037

[28] Dehnert M Fingerle V Klier C Talaska T Schlaud M Krause G Seropositivity of Lyme borreliosis and associated risk factors: a population-based study in Children and Adolescents in Germany (KiGGS). PLoS One. 2012;7(8):e41321. 10.1371/journal.pone.0041321 22905101 PMC3419690

[29] Kiewra D Zaleśny G Relationship between temporal abundance of ticks and incidence of Lyme borreliosis in Lower Silesia regions of Poland. J Vector Ecol. 2013;38(2):345-52. 10.1111/j.1948-7134.2013.12050.x 24581365

[30] Schwarz A Hönig V Vavrušková Z Grubhoffer L Balczun C Albring A Abundance of Ixodes ricinus and prevalence of Borrelia burgdorferi s.l. in the nature reserve Siebengebirge, Germany, in comparison to three former studies from 1978 onwards. Parasit Vectors. 2012;5(1):268. 10.1186/1756-3305-5-268 23171708 PMC3523962

[31] van den Wijngaard CC Hofhuis A Harms MG Haagsma JA Wong A de Wit GA The burden of Lyme borreliosis expressed in disability-adjusted life years. Eur J Public Health. 2015;25(6):1071-8. 10.1093/eurpub/ckv091 26082446

[32] van den Wijngaard CC Hofhuis A Wong A Harms MG de Wit GA Lugnér AK The cost of Lyme borreliosis. Eur J Public Health. 2017;27(3):538-47. 10.1093/eurpub/ckw269 28444236

[33] European Centre for Disease Prevention and Control (ECDC), European Food Safety Authority (EFSA). VectorNet: A European network for sharing data on the geographic distribution of arthropod vectors, transmitting human and animal disease agents. Stockholm: ECDC. [Accessed 27 Dec 2016]. Available from: http://ecdc.europa.eu/en/healthtopics/vectors/VectorNet/Pages/VectorNet.aspx

[34] Wormser GP Dattwyler RJ Shapiro ED Halperin JJ Steere AC Klempner MS The clinical assessment, treatment, and prevention of lyme disease, human granulocytic anaplasmosis, and babesiosis: clinical practice guidelines by the Infectious Diseases Society of America. Clin Infect Dis. 2006;43(9):1089-134. 10.1086/508667 17029130

[35] Kullberg BJ Berende A van der Meer JW The challenge of Lyme disease: tired of the Lyme wars. Neth J Med. 2011;69(3):98-100. 21444933

[36] Berende A ter Hofstede HJ Donders AR van Middendorp H Kessels RP Adang EM Persistent Lyme Empiric Antibiotic Study Europe (PLEASE)--design of a randomized controlled trial of prolonged antibiotic treatment in patients with persistent symptoms attributed to Lyme borreliosis. BMC Infect Dis. 2014;14(1):543. 10.1186/s12879-014-0543-y 25318999 PMC4203907

[37] Berende A ter Hofstede HJ Vos FJ van Middendorp H Vogelaar ML Tromp M Randomized Trial of Longer-Term Therapy for Symptoms Attributed to Lyme Disease. N Engl J Med. 2016;374(13):1209-20. 10.1056/NEJMoa1505425 27028911

[38] World Health Organization (WHO). Immunization, Vaccines and Biologicals. Sentinel Surveillance Geneva: WHO. [Accessed 27 Dec 2016]. Available from: http://www.who.int/immunization/monitoring_surveillance/burden/vpd/surveillance_type/sentinel/en/

[39] Krause G Ropers G Stark K Notifiable disease surveillance and practicing physicians. Emerg Infect Dis. 2005;11(3):442-5. 10.3201/eid1103.040361 15757561 PMC3298248

[40] Jelastopulu E Merekoulias G Alexopoulos EC Underreporting of communicable diseases in the prefecture of Achaia, western Greece, 1999-2004 - missed opportunities for early intervention. Euro Surveill. 2010;15(21):19579. 20519103 10.2807/ese.15.21.19579-en

[41] Richard JL Vidondo B Mäusezahl M A 5-year comparison of performance of sentinel and mandatory notification surveillance systems for measles in Switzerland. Eur J Epidemiol. 2008;23(1):55-65. 10.1007/s10654-007-9187-1 17899399

[42] Gibbons CL Mangen MJ Plass D Havelaar AH Brooke RJ Kramarz P Measuring underreporting and under-ascertainment in infectious disease datasets: a comparison of methods. BMC Public Health. 2014;14(1):147. 10.1186/1471-2458-14-147 24517715 PMC4015559

[43] Braks M Medlock JM Hubalek Z Hjertqvist M Perrin Y Lancelot R Vector-borne disease intelligence: strategies to deal with disease burden and threats. Front Public Health. 2014;2:280. 10.3389/fpubh.2014.00280 25566522 PMC4273637

